# Supplementary material for: Unravelling reference bias in ancient DNA datasets
Source: Bioinformatics. 2024 Jul 3;40(7):btae436. doi: 10.1093/bioinformatics/btae436 (PMC11254355; doi:10.1093/bioinformatics/btae436)
Supplement: btae436_Supplementary_Data [file btae436_supplementary_data.zip › S1-S4_data_plots.zip!/S1-S4_data/rhino_sim_plots.zip!/rhino_sim_plots/verysensitive_bowtie_rhino_div1_10end_by_MQ.pdf]

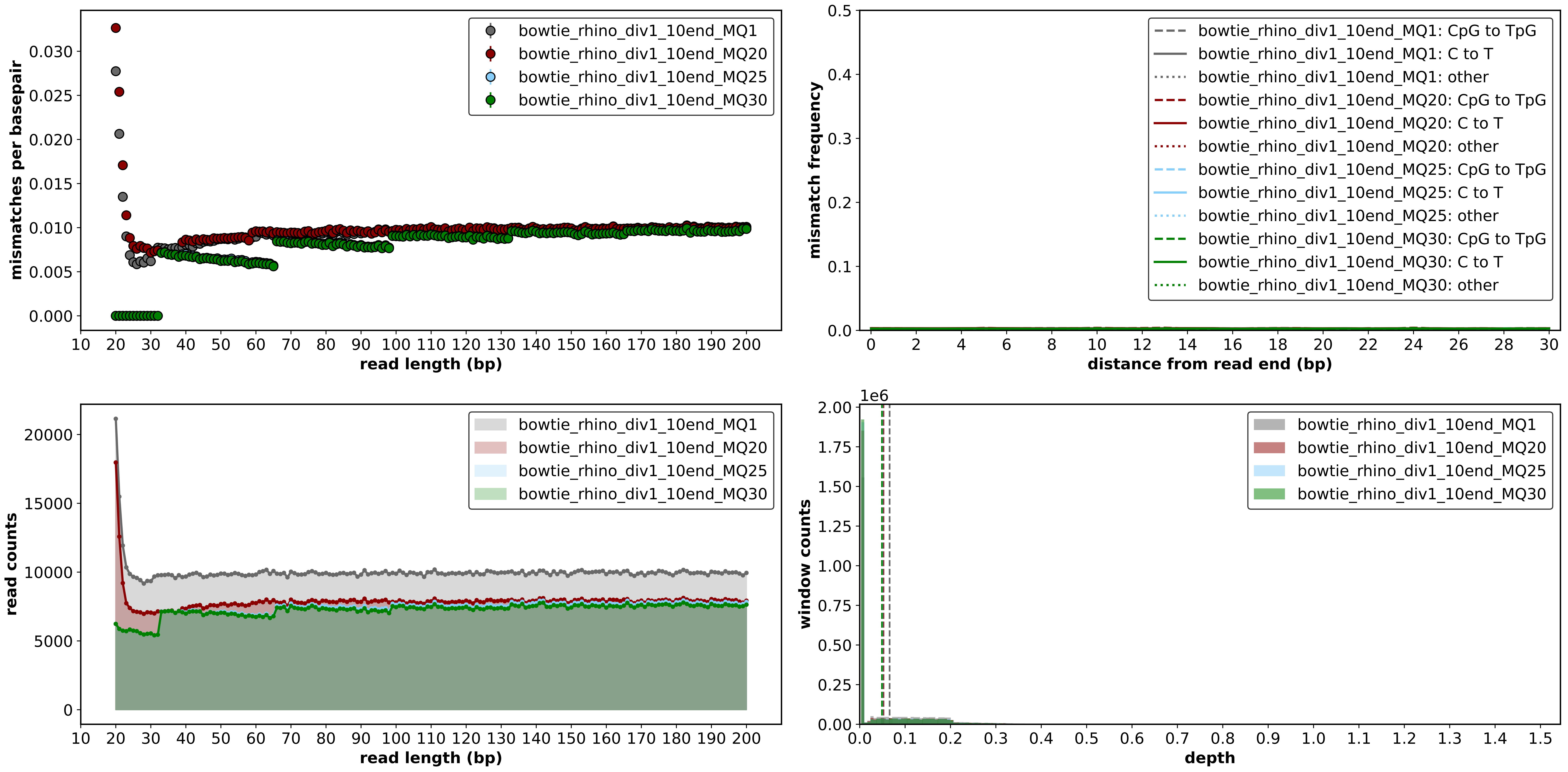

mismatches per basepair
0.000
0.005
0.010
0.015
0.020
0.025
0.030
read length (bp)
10 20 30 40 50 60 70 80 90 100 110 120 130 140 150 160 170 180 190 200
bowtie_rhino_div1_10end_MQ1
bowtie_rhino_div1_10end_MQ20
bowtie_rhino_div1_10end_MQ25
bowtie_rhino_div1_10end_MQ30
mismatch frequency
0.0
0.1
0.2
0.3
0.4
0.5
distance from read end (bp)
0 2 4 6 8 10 12 14 16 18 20 22 24 26 28 30
bowtie_rhino_div1_10end_MQ1: CpG to TpG
bowtie_rhino_div1_10end_MQ1: C to T
bowtie_rhino_div1_10end_MQ1: other
bowtie_rhino_div1_10end_MQ20: CpG to TpG
bowtie_rhino_div1_10end_MQ20: C to T
bowtie_rhino_div1_10end_MQ20: other
bowtie_rhino_div1_10end_MQ25: CpG to TpG
bowtie_rhino_div1_10end_MQ25: C to T
bowtie_rhino_div1_10end_MQ25: other
bowtie_rhino_div1_10end_MQ30: CpG to TpG
bowtie_rhino_div1_10end_MQ30: C to T
bowtie_rhino_div1_10end_MQ30: other
read counts
0
5000
10000
15000
20000
read length (bp)
10 20 30 40 50 60 70 80 90 100 110 120 130 140 150 160 170 180 190 200
bowtie_rhino_div1_10end_MQ1
bowtie_rhino_div1_10end_MQ20
bowtie_rhino_div1_10end_MQ25
bowtie_rhino_div1_10end_MQ30
window counts
1e6
0.00
0.25
0.50
0.75
1.00
1.25
1.50
1.75
2.00
depth
0.0 0.1 0.2 0.3 0.4 0.5 0.6 0.7 0.8 0.9 1.0 1.1 1.2 1.3 1.4 1.5
bowtie_rhino_div1_10end_MQ1
bowtie_rhino_div1_10end_MQ20
bowtie_rhino_div1_10end_MQ25
bowtie_rhino_div1_10end_MQ30
